# Regulation of Energy Metabolism and Anti-Inflammatory Activities of Mastiha Fractions from *Pistacia lentiscus* L. *var. chia*

**DOI:** 10.3390/foods12071390

**Published:** 2023-03-24

**Authors:** Foteini D. Kalousi, Federica Pollastro, Aikaterini G. Karra, Ioannis Tsialtas, Achilleas Georgantopoulos, Stefano Salamone, Anna-Maria G. Psarra

**Affiliations:** 1 Department of Biochemistry and Biotechnology, University of Thessaly, Biopolis, 41500 Larissa, Greece; fokalous@uth.gr (F.D.K.); aikaterini.g.karra@gmail.com (A.G.K.); tsialtasj@gmail.com (I.T.); ageorgant@uth.gr (A.G.); 2 Department of Pharmaceutical Sciences, University of Eastern Piedmont, 28100 Novara, Italy; federica.pollastro@uniupo.it (F.P.); stefano.salamone@unipo.it (S.S.); 3 PlantaChem Srls, Via Amico Canobio, 28100 Novara, Italy

**Keywords:** Chios Mastiha, glucocorticoid receptor, anti-inflammatory action, apoptotic action, triterpenes, AMPK, PEPCK

## Abstract

*Pistacia lentiscus* L. *var. chia* resin (Chios Mastiha), the first natural chewing gum, is widely used in Mediterranean cuisine and has been used in traditional medicine from ancient times. Regarding its chemical composition, Chios Mastiha is known to be rich in triterpenes. Triterpenes have a similar structure to glucocorticoids (GCs), the steroid hormones that exert strong anti-inflammatory activities and play crucial roles in the regulation of cellular metabolism. To simplify the characterization of the bioactive compounds of Mastiha resin, three different polarity fractions were isolated and were further analyzed regarding their main chemical composition and an assessment of their biological activities. The biological assessment focused on the evaluation of the potential anti-proliferative, anti-inflammatory, and apoptotic activities as well as the possible interference of the three different polarity Mastiha fractions with the glucocorticoid receptor signaling, with the aim of characterizing the biochemical mechanisms of the actions of the Mastiha fraction. Applying MTT cell viability assay, luciferase/β-galactosidase assay, and Western blot analysis showed that Chios Mastiha apolar, medium-polar, and polar fractions reduced the HEK293 cell viability in a dose-dependent manner, possibly by mitochondrial-mediated induction of apoptosis. Medium-polar and polar Mastiha fractions also suppressed the GR and NF-κΒ transcriptional activation and the p65 protein levels. These activities were accompanied by the modulation of protein levels of regulatory molecules playing a crucial role in cellular energy homeostasis, such as GR, phosphoenolpyruvate carboxykinase (PEPCK), and/or peroxisome proliferator-activated receptor alpha (PPARα), and by the induction of phosphorylation and the activation of the AMP-activated protein kinase (AMPK). The medium-polar fraction was found to be enriched in triterpenes, such as lupeol, 24Z-masticadienonic acid methyl ester, and 24Z-isomasticadienonic acid methyl ester, and it was the most active one, so we propose that triterpenes in medium-polar fraction are possibly the bioactive compounds responsible for Mastiha’s regulatory actions on energy metabolism and anti-inflammatory activities via interference with GR, NF-κΒ, and AMPK signaling. This highlights its potential applications in many fields of pharmaceutical, cosmetic, and nutraceutical interest.

## 1. Introduction

Chios Mastiha is the resin obtained from the *Pistacia lentiscus* L. *var. chia* (Mastiha tree), which is an endemic plant cultivated exclusively in the Southern part of the Greek Island of Chios. Chios Mastiha is the first natural chewing gum and is widely used in traditional Mediterranean cooking and beverages due to its distinctive aroma and taste. Mastiha is also an important dietary supplement, especially in cases of a lack of trace elements [1]. It has a wide application in perfumery, dentistry, and cosmetics, too [2]. Moreover, over 2500 years, it has been used in Greek traditional medicine from Dioscurides to Galenus in order to cure gastrointestinal inflammatory disorders, such as peptic ulcers [3]. In recent years, many in vitro and clinical studies have revealed a wide variety of the biological properties of Chios Mastiha, such as anti-inflammatory, antioxidant [1,4], anti-microbial, hypolipidemic, and anti-cancer ones. In 2015, Chios Mastiha has been recognized by the European Medicine Agency (EMA) as a traditional herbal remedy for minor dyspeptic disorders and skin inflammation treatments [5].

The composition of Chios Mastiha has been widely studied, revealing that 25–30% of crude Mastiha consists of a sticky polymer β-myrcene, while triterpenes represent approximately 65–70% of its composition. Thus, triterpenes such as moronate, oleanolate, oleanonate, masticadienonate, masticadienolate [6,7,8], and the triterpenic acids named 24Ζ-masticadienonic acid and 24Ζ-masticadienolic acid [3] have all been identified in high abundance in the acidic fraction of Chios Mastiha. Similarly, in the neutral fraction of Chios Mastiha, triterpenes such as 28-norolean-17-en-3-one, oleanonic aldehyde, β-amyrone, dammaradienone, norlupenone, isomasticadienolic aldehyde [7,8], tirucallol, oleanonic aldehyde, and oleanolic aldehyde have been identified [3] in high concentrations. Moreover, a chemical composition analysis of Chios Mastiha has revealed the presence of many phenolic and flavonoid compounds, such as gallic acid, tyrosol, benzoic acid, phenylacetic acid, caffeic acid, vanillic acid, O-coumaric acid, cinnamic acid, oleanolic acid, and ursolic acid [9,10].

Natural glucocorticoids (GCs) like cortisol are steroid hormones, which are being secreted from the adrenal glands, and which exert their actions through the glucocorticoid receptor (GR). These hormones are essential for many physiological processes, such as metabolism, growth, development, immunological responses, apoptosis, and mental health [11]. The glucocorticoid receptor localizes mainly in the cytosol in an inactive state. The binding of the hormone to GR induces GR conformational changes, which lead to GR nuclear translocation and the activation or suppression of its target gene expression [12,13]. Synthetic glucocorticoids, such as dexamethasone, constitute the most frequent prescribed medication to cure inflammatory and immune diseases due to their strong anti-inflammatory and immunosuppressive activities. However, the prolonged and high doses of glucocorticoid treatment can cause several side effects, such as diabetes, muscle atrophy, osteoporosis, hypertension, and peptic ulcers [14]. This gives rise to research activities focusing on the development of selective glucocorticoid receptor activators to optimize the beneficial properties of GCs and minimize their harmful side effects [15].

In this context, plant origin compounds, such as triterpenoids, have raised the interest of many researchers due to their structural similarities with glucocorticoids and their promising biological properties, pointing out their potential as selective glucocorticoid receptor modulators. Experimental results from our and, also, from other groups have revealed that plant origin triperpenoids, such as echinocystic acid and its 3-O-glucoside derivative [16], protopanaxadiol, protopanaxatriol [17,18], boswellic acid alpha, and beta, as well as its derivatives such as acetyl-11-keto beta boswellic acid, 11-keto beta boswellic acid, 3-O-acetyl boswellic acid alpha, and 3-O-acetyl boswellic acid beta, can induce translocation of GR to the nucleus and the activation of its transrepressional function [19] with no GR transactivation. This indicates their potential as lead molecules for the design and production of selective GR modulators. Our previous studies also showed that the medium-polar and the polar fractions from *Pistacia lentiscus* L. *var. chia* leaves interfere with GR signaling via repression of GR transcriptional activation and the reduction of its protein levels in a proteasomal-dependent manner [20].

In light of this, and considering the enrichment of Mastiha in triterpenoids, in this study we investigated the biological activities of different polarity fractions isolated from the resin of the Chios Mastiha tree. Emphasis was given to activities related to the actions of GCs. Thus, the possible anti-inflammatory and anti-proliferative properties, and the interference with the regulation of apoptosis, GR-transactivation and -transrepression, and GR-associated metabolic activities of three different polarity Mastiha fractions (apolar, medium-polar, and polar) were evaluated, applying Western blot analysis, MTT assays, and/or luciferase reporter gene assays in the GR-positive human HEK293 cell line.

## 2. Materials and Methods

### 2.1. Chemicals

Dulbecco’s modified Eagle medium (DMEM) (4.5 g/L glucose), fetal bovine serum (FBS), and molecular weight protein markers were obtained from Thermo Fischer Scientific (GmbH, Frankfurt, Germany). Recombinant Human Tumor Necrosis Factor alpha (TNFα) was obtained from Peprotech (London, UK). A cocktail of protease inhibitors was obtained from Roche (Mannheim, Germany). Reporter lysis buffer and luciferin were purchased from Promega Corporation (Madison, WI, USA). For chromatography studies, silica gel 60, Celite^®^ 545 particle size 0.02–0.1 mm, CAS 68855-54-9, pH 10 (100 g/L, H_2_O, 20 °C), neutral alumina Alugram^®^, and RP C-18 silica gel were used and obtained from Macherey-Nagel (Düren, Germany). 1 H (400 MHz) spectra were measured on Bruker 400 NMR spectrometers. Chemical shifts were referenced to the residual solvent signal (CDCl3: δH = 7.26). Purifications were monitored by TLC on Merck 60 F254 (0.25 mm) plates, visualized by staining with 5% H_2_SO_4_ in EtOH and heating. HPLC JASCO Hichrom, 250 × 25 mm, silica UV−vis detector-2075 plus (Oklahoma, Japan) was used. Dexamethasone (DEX) and all the other solvents and chemicals used were obtained from Sigma-Aldrich (St. Louis, MO, USA). Finally, the Chios Mastiha was a kind donation from the Mastiha Shop and the Chios Mastic Growers Association.

### 2.2. Chios Mastiha Fractionation

Resin from *Pistacia lentiscus* L. (10 g) was dissolved in acetone (volume acetone/plant material weight, 10:1) upon stirring for 2 h. Filtration of the suspension was followed to get rid of the insoluble material and to provide 9.5 g (95%) of a yellow, creamy acetonic extract after evaporation of the solvent.

A minimum amount of acetone was then used to dissolve the dried extract and, subsequently, silica gel was added (1:3 weight/weight g). This latter suspension was completely evaporated at reduced pressure. The powder obtained after evaporation was stratified on a Celite layer, as previously described [20]. Three different polarity fractions were then isolated by the addition of solvents of increasing polarity (petroleum ether (Pe), ethyl acetate (EtOAc), and tetrahydrofuran (THF)), as previously described [20]. The three vacuum filtrates were collected and evaporated to obtain 1.91 g of apolar fraction (ap) (20.1% yield, Pe extract), 5.81 g of medium-polar fraction (mp) (61.2% yield, EtOAc extract), and 0.56 g of polar fraction (p) (5.9% yield, THF extract).

### 2.3. Chemical Characterization of Different Polarity Mastiha Fractions

**Figure 1:**
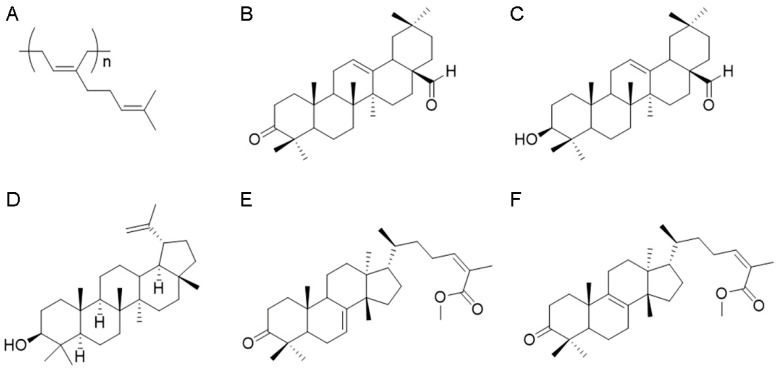
Phytochemical characterization of *Pistacia lentiscus* L. resin. Chemical structures of 1,4-poly-β-myrcene (**A**), keto-oleanolic aldehyde (**B**), oleanolic aldehyde (**C**), lupeol (**D**), 24Z-masticadienonic acid methyl ester (**E**), and 24Z-isomasticadienonic acid methyl ester (**F**).

The apolar fraction, 1.91 g of acetonic extract from *P. lentiscus* resin, was purified by applying low-pressure chromatography analysis on silica gel (95 g, petroleum ether-EtOAc gradient from 100 to 70:30) to provide 235.5 mg of the polymer 1,4-poly-β-myrcene (Figure 1A), 418.2 mg of the keto-oleanolic aldehyde (Figure 1B), and 152.3 mg of the oleanolic aldehyde (Figure 1C). The compounds were identified with ^1^H NMR analysis according to the literature [21,22].

Next, 5.81 g of the medium-polar fraction from acetonic resin extract was fractionated, applying chromatography analysis on silica gel (300 g, petroleum ether-EtOAc gradient from 80:20 to 50:50) to afford, once again, 261.5 mg of the oleanolic aldehyde (Figure 1C), 218.5 mg of lupeol (Figure 1D) [23], and 341.3 mg of a mixture of triterpenoid acids. To purify this latter fraction, the triterpenoid acids were esterified with TMSiCHN2 (1 mL, 2.0 M, MW 114.2 g/Mol) in MeOH (4 mL) and diethyl ether (4 mL). The course of the reaction was followed by TLC (petroleum ether-EtOAc 80:20). After stirring for 1 h at room temperature, the solvent was evaporated under reduced pressure and the residue purified with HPLC on silica (petroleum ether-EtOAc gradient from 90:10 to 80:20) to give 49.9 mg of 24Z-masticadienonic acid methyl ester (Figure 1E) and 69.9 mg of 24Z-isomasticadienonic acid methyl ester (Figure 1F). Identification of these compounds was achieved according to previous publications [1,24,25,26].

The polar fraction (0.56 g) of acetonic extract of *P. lentiscus* resin has been analyzed by ^1^H NMR spectra, revealing the presence of phenolic compounds and the lack of triterpenoids.

### 2.4. Antibodies

Mouse monoclonal antibodies against glyceraldehyde-3-phosphate dehydrogenase (GAPDH), human glucocorticoid receptor (GR), peroxisome proliferator-activated receptor alpha (PPARα), and rabbit polyclonal antibodies against p65 subunit of NF-κB and phosphoenolpyruvate carboxykinase (PEPCK) were purchased from Santa Cruz Biotechnology (Europe Inc., Heidelberg, Germany). Monoclonal antibodies against β-actin (Sigma Aldrich, St. Louis, MO, USA), procaspase-9 (Cell Signaling Technology, Leiden, The Netherlands), and rabbit polyclonal antibodies against bcl-2, AMPKα, phosphorylated AMPKα at Thr172 (pAMPKαThr172), and procaspase-3 (Cell Signaling Technology) were also used.

### 2.5. Cell Culture

The human embryonic kidney 293 (HEK293) cell line was purchased from the American type culture collection (ATCC). HEK293 cells were used because of their high efficiency in transfection experiments and their endogenous GR expression. HEK293 cells were cultured at 37 °C and 5% CO_2_ humidity, in DMEM, supplemented with 10% FBS, 2 mM L-glutamine, and 100 units/mL penicillin/streptomycin (pen/strep). 48 h before the addition of resin fractions, cells were cultured in DMEM medium free of phenol red and supplemented with 10% charcoal stripped FBS (CSF), 2 mM L-glutamine, and 100 units/mL pen/strep.

### 2.6. MTT Cell Viability Assay

HEK293 cells were plated in 96-well plate at a density of 1.5 × 10^4^ cells/well for 24 h in DMEM, supplemented with 10% FBS, 2 mM L-glutamine, and 100 units/mL pen/strep, as previously described [20]. Cells were then treated with resin fractions diluted in 100% DMSO. The final concentration of the fractions was 5–100 μg/mL. DMSO treatment (1:1000) was used as control condition. After 6, 24, or 48 h treatment, 0.5 mg/mL MTT (diluted in PBS 1X) was added for 3–4 h. Subsequently, the produced crystals of formazan were diluted in 100% isopropanol upon shaking. Finally, absorbance was measured at 570 nm (multimode plate reader, EnSpire, PerkinElmer, Beaconsfield, UK). Moreover, background absorbance was measured at 690 nm, as reference.

### 2.7. GR and NFκΒ Transcriptional Activity Measurement

GR and Nuclear factor-kappa B (NF-κB) transcriptional activation was evaluated, applying luciferase reporter gene assay, as previously described [20]. Briefly, HEK293 cells were plated on 24-well plates, at a density of 3 × 10^4^ cells/well, in no phenol red DMEM medium supplemented with 10% CSF, 2 mM L-glutamine, and 100 units/mL pen/strep. After 24 h, cells were transiently co-transfected using calcium phosphate, with a Glucocorticoid response elements (GRE) or a NF-κΒ response elements (NF-κB-RE) promoter-driven luciferase construct and a β-galactosidase reporter construct. At 14–16 h upon transfection, the medium was replaced with fresh growth medium. After 24 h, cells were triggered either by 1 μM DEX diluted in EtOH or 20 ng/mL TNFα diluted in ddH_2_O for the evaluation of GR or NF-κB activity, respectively, with or without the presence of increasing concentrations of Chios Mastiha fractions for 6 h. DMSO/EtOH treatment (1:1000) was used as control condition. Subsequently, reporter lysis buffer was used for the lysis of the cells. Finally, a chemiluminometer (LB 9508, Berthhold) was used to measure the enzymatic activities of the produced luciferase and β-galactosidase in the cell extracts. β-galactosidase activity measurement was assessed to normalize luciferase activity (RLU).

### 2.8. Western Blot Analysis

HEK293 cells were plated on 6 well plates at a density of 2 × 10^5^ cells/well in DMEM medium without phenol red supplemented with 10% CSF, 2 mM L-glutamine, and 100 units/mL pen/strep for 48 h. Cells were then treated with the indicated amounts of Chios Mastiha fractions for an additional 24 or 48 h, with or without 10nM DEX. DMSO/EtOH treatment (1:1000) was used as control condition. Cells were then washed in PBS 1X, lysed in lysis buffer A (20 mM Tris pH:7.5, 250 mM NaCl, 3 mM EDTA, 0.5% Triton), and supplemented with cocktail protease inhibitors, DTT, and PMSF. Bradford assay was then applied for protein measurement. Subsequently, cell extracts were electrophoresed in discontinuous SDS-PAGE and Western blotted using specific antibodies against PEPCK, GR, PPARα, p65, AMPKα, pAMPKαThr172, procaspase-9, procaspase-3, and bcl-2, as previously described [20]. β-actin or GAPDH protein levels were evaluated for the normalization of the results. Enhanced chemiluminescence was applied for the detection of the protein bands. Image J (1.52 p) analysis (NIH, Bethesda, MD, USA) was applied for the quantification of the bands’ intensity. The bands’ intensity of each molecule were normalized against the respective bands’ intensity of β-actin, or GAPDH, to give the relative protein levels of each protein analyzed. Relative protein levels in control (vehicle-treated) cells were set as 1.

### 2.9. Statistical Analysis

All results are expressed as mean ± SD. Data were analyzed by an independent *t*-test or by a One-Way analysis of variance (ANOVA) or a Two-Way ANOVA, followed by Tukeys’s post hoc test using SPSS or Stat Plus software, respectively. Differences were considered significant at a two tailed *p*-value < 0.05.

## 3. Results

### 3.1. Chemical Characterization of Chios Mastiha Fractions

In this study, with the aim of simplifying the analysis of the bioactive compounds, a fractionation protocol was applied to the acetonic extract from *P. lentiscus* resin to obtain three Mastiha fractions of different polarity. Following this procedure, the apolar fraction corresponds to a neutral fraction, while acid triterpenoids were concentrated in the medium-polar fraction. In this frame, we followed the purification, looking for triterpenoids in the apolar and the medium-polar fractions. The polar fraction, instead, was analyzed only by ^1^H NMR to achieve a qualitative characterization of the major compounds.

The apolar fraction (ap) of Mastiha is concentrated in the 1,4-poly-β-myrcene (Figure 1A and Figure S1) detectable from the typical presence of protons on double bounds overlapping at H = 5.14 ppm, the allylic protons at H = 2.05 ppm, and the methyl moieties, respectively, at H = 1.61 and H = 1.69 ppm. In this fraction, triterpenoids are represented by the keto-oleanolic aldehyde (Figure 1B and Figure S2) and the oleanolic aldehyde (Figure 1C and Figure S3), in which the aldehyde moiety is clearly visible at H = 9.39 ppm for both compounds B and C, and compound C presents the assignments at H = 3.23–3.19 ppm typical of the hydroxylic group that is missing in the keto-oleanolic aldehyde due to the ketonic oxidation.

As expected, the most abundant yield of triterpenoid acids is in the medium-polar (mp) fraction, in which lupeol (Figure 1D and Figure S4) has been identified together with a residual amount of oleanolic aldehyde and the acid triterpenoids, purified after methylation reaction, which leads to the 24Z-masticadienonic acid methyl ester (Figure 1E and Figure S5) and 24Z-isomasticadienonic acid methyl ester (Figure 1F and Figure S6). Compounds E and F are recognizable due to the esterification reaction of the carboxylic group (H = 3.74 and 3.73 ppm) that simplified the HPLC purification. Moreover, compound F is characterized by the presence of the allylic proton at H = 5.96 ppm, whereas compound E has the additional signals at H = 5.31 ppm.

The ^1^H NMR analysis of the polar (p) fraction revealed the presence of phenolic compounds as demonstrated by chemical shifts between H = 7.26–7.57 ppm (Figure S7).

### 3.2. Effect of Mastiha Fractions on Viability of HEK293 Cells

**Figure 2:**
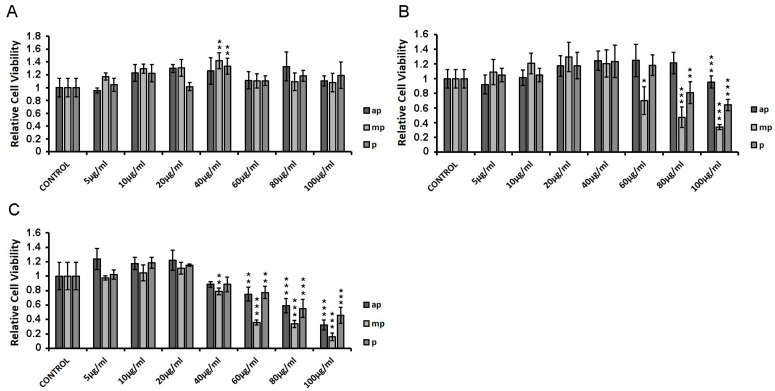
HEK293 cell viability measurement in the presence of Chios Mastiha apolar (ap), medium-polar (mp), and polar (p) fractions, upon 6 h (**A**), 24 h (**B**), and 48 h (**C**) treatment. Cell viability was evaluated by MTT assay. DMSO (1:1000) was added in control condition. Relative cell viability is expressed as the viability of the cells treated with the respective resin fractions compared to the cell viability of the DMSO-treated cells. Cell viability of control cells was set at 1. Data are expressed as the mean ± SD, (n = 3–9), * *p* < 0.05; ** *p* < 0.01; *** *p* < 0.001.

HEK293 cells were incubated with increasing concentrations of apolar, medium-polar, and polar resin fractions from *Pistacia lentiscus* L. *var. chia* (Chios Mastiha) for 6 h (Figure 2A), 24 h (Figure 2B), and 48 h (Figure 2C), and cell viability was assessed by applying MTT assay. As shown in Figure 2A, upon 6 h treatment, no cytotoxicity was observed. Upon 24 h treatment, cytotoxic effects of the Chios Mastiha fractions were observed at concentrations higher than 60 μg/mL. The medium-polar (mp) fraction was the most cytotoxic. The mp fraction caused up to 65% decrease in cell viability at the concentrations of 60 to 100 μg/mL. The polar fraction showed up to 35% reduction in viability of HEK293 cells at concentrations of 80–100 μg/mL. On the contrary, the apolar fraction caused the least reduction in cell viability at 4.5% in a concentration of 100 μg/mL (Figure 2B). As shown in Figure 2C, the highest cytotoxicity was observed upon 48 h treatment, with the medium-polar fraction being the most cytotoxic. A 20%–80% reduction in cell viability was noticed at concentrations from 40 to 100 μg/mL. The apolar and the polar Mastiha fractions caused statistically significant reductions in the viability of the HEK293 cells up to 67% and 54%, respectively, at concentrations from 60 to 100 μg/mL. No cytotoxicity of the apolar and the polar fractions was observed at concentrations up to 40 μg/mL, whereas a 20% decrease in the viability of HEK293 cells was observed by the medium-polar Mastiha fraction at the same concentration (40 μg/mL).

### 3.3. Suppression of the DEX-Induced GR Transcriptional Activation by the Chios Mastiha Fractions

**Figure 3:**
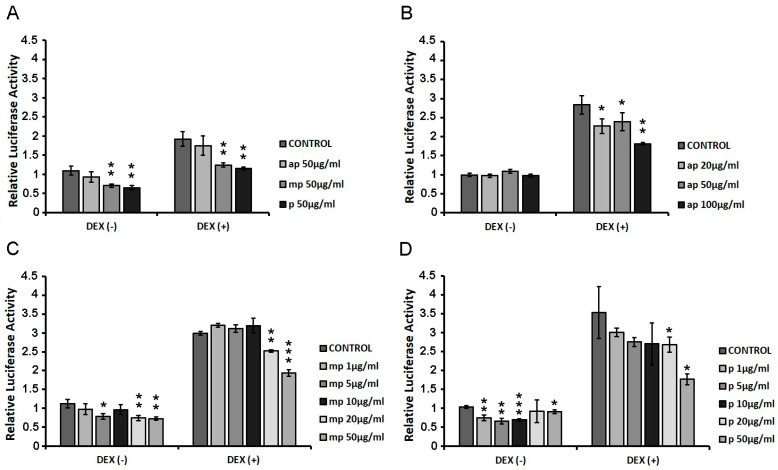
Suppression of the DEX-induced GR transcriptional activation by the Chios Mastiha different polarity fractions. Luciferase and β-galactosidase enzymatic activity was evaluated in extracts from HEK293 cells cultured in the absence of hormones, transiently co-transfected with a GREs-Luc reporter gene construct and a β-galactosidase reporter construct, and subsequently subjected to treatment with (**A**) 50 μg/mL apolar (ap), medium-polar (mp), and polar (p) Mastiha fractions (**B**) 20–100 μg/mL apolar (ap) Mastiha fraction, (**C**) 1–50 µg/mL medium-polar (mp) Mastiha fraction, and (**D**) 1–100µg/mL polar (p) Mastiha fraction and/or 1 μΜ DEX, for 6 h. DMSO (1:1000) and EtOH (1:1000) were added in control condition. Relative luciferase activity results from normalization of the luciferase activity to β-galactosidase activity. Relative luciferase activity of control cells was set as 1. Data are expressed as the mean ± SD, (n = 2–3), * *p* < 0.05; ** *p* < 0.01; *** *p* < 0.001.

HEK293 cells were transiently co-transfected with GREs and β-galactosidase constructs as described in the Materials and Methods section, and they were treated with increasing concentrations of ap, mp, and p resin fractions from *Pistacia lentiscus* L. *var. chia* (1 μg/mL–100 μg/mL) in the presence or absence of 1 µM DEX for 6 h. Applying luciferase reporter gene assay, the effect of the fractions on GR transcriptional activation was assessed. As shown in Figure 3A, the first screening of the three fractions (50 μg/mL), showed that the medium-polar and the polar ones were the most active, reducing the GR transcriptional activation up to 40% with or without DEX compared to control DMSO-treated cells. We then proceeded with the assessment of the effect of the apolar fraction at a range concentration of 20–100 μg/mL (Figure 2B) and that of the medium-polar and the polar fractions (1–50 μg/mL) (Figure 3C,D). As shown in Figure 3B, the apolar fraction caused a statistically significant suppression in the DEX-induced GR transcriptional activation by approximately 20 and 35% at concentrations of 20 and 100 μg/mL, respectively. A total of 20 μg/mL and 50 μg/mL of the medium-polar fraction suppressed the DEX-induced GR transcriptional activation by approximately 15 and 35%, respectively (Figure 3C), while the polar fraction caused a statistically significant reduction by up to 50% at concentration of 50 μg/mL (Figure 3D), being the most active one. The medium-polar and the polar Mastiha fractions reduced the GR transcriptional activation up to 35%, even without co-administration of DEX.

**Figure 4:**
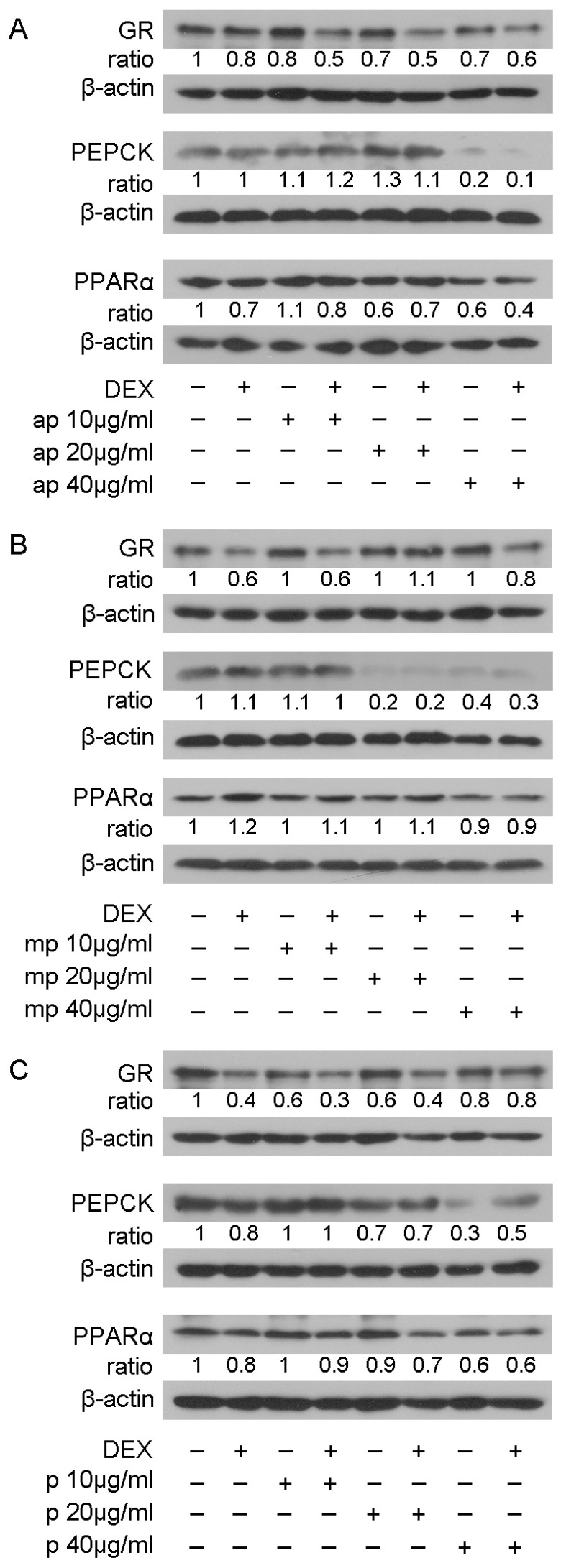
Regulation of GR, PEPCK, and PPARα protein levels by Chios Mastiha different polarity fractions. Representative images from Western blot analysis of GR, PEPCK, and PPARα from HEK293 cell extracts precultured in hormone-free medium for 48 h and subsequently treated with 10, 20, and 40 μg/mL of apolar (**A**), medium-polar (**B**), and polar (**C**) fractions for 48 h. Ratios result from normalization of GR, PEPCK, and PPARα bands intensity to the respective β-actin ones. Relative protein levels in control cells were set as 1.

The suppressive effect of the Chios Mastiha fractions on GR transcriptional activation was further investigated by assessing the effect of Mastiha fractions on the protein levels of GR and its target genes PEPCK and PPARα [27,28], applying Western blot analysis. Thus, HEK293 cells were treated with 10, 20, and 40 μg/mL of Mastiha fractions, with or without the addition of 10 nM DEX or DMSO/EtOH for 24 h (Figure S8) and 48 h (Figure 4). Upon 24 h treatment, no remarkable changes on PEPCK and PPARα protein levels were observed (Figure S8). Thus, we proceeded to 48 h treatment (Figure 4), with GR protein levels being reduced up to 30% and 40% upon treatment with the apolar (Figure 4A) and polar (Figure 4C) fractions, respectively, at a concentration of 10–40 μg/mL. An additive 10–30% decrease in GR protein levels was observed when fractions co-administrated with DEX. Medium-polar fraction did not cause any effect on GR protein levels. As far as the effects on PEPCK protein levels are concerned, the medium-polar fraction caused an 80% reduction even at a concentration of 20 μg/mL (Figure 4B), being the most active one, while 40 μg/mL apolar and polar fractions reduced PEPCK protein levels by up to 80% and 70%, respectively (Figure 4A,C). As far as PPARα protein levels, the apolar and polar fractions caused 40% reduction at concentrations 20 and 40 μg/mL, respectively. A slight reduction in PPARα protein levels was also detected in the presence of the medium-polar fraction at the highest concentration examined (40 μg/mL).

**Figure 5:**
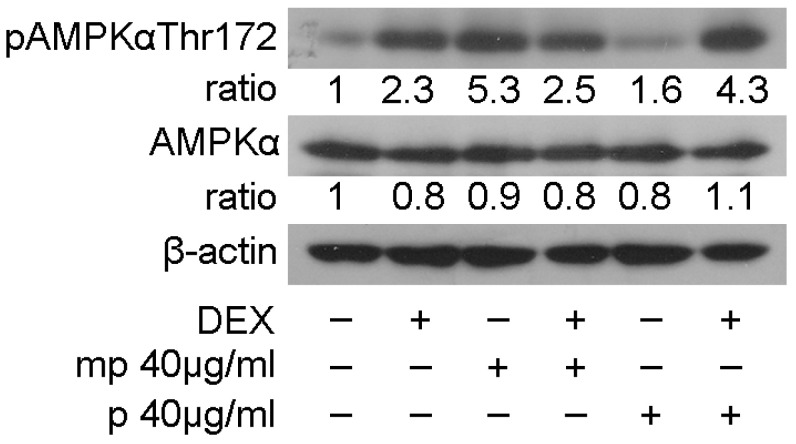
Chios Mastiha fractions cause phosphorylation of AMPKα protein levels. Western blot analysis of β-actin, AMPKα, and phosphorylated AMPKα protein levels in HEK293 cell extracts precultured in hormone-free medium for 48 h and subsequently treated with 40 μg/mL Mastiha fractions for 45 min. Ratios result from normalization of pAMPKαThr172 and AMPKα bands intensity to the respective β-actin ones. Relative protein levels in control cells were set as 1.

The most active fractions, as regards suppression in GR transcriptional activation, mp, and p, and their effect on the regulation in GR, PEPCK, and PPARα protein levels prompted us to examine the effect of medium-polar and polar Mastiha fractions on the regulation of AMP-activated protein kinase (AMPK) activity. AMPK regulates cellular metabolism through energy homeostasis balance. Specifically, AMPK senses cellular energy deprivation. Elevated AMP levels cause AMPK phosphorylation and its sequential activation. Activated AMPK, then, promotes catabolic procedures to provide energy supply for the cells [29]. GCs are also well-known catabolic hormones [30]. Moreover, previous studies have shown cross-talk of AMPK and GR signaling. Specifically, activated AMPK causes regulation of GR transcriptional activation through induction of GR phosphorylation via an indirect manner [31]. To evaluate Mastiha fractions’ effects on the regulation of AMPK activity, 40 μg/mL of the medium-polar and the polar fractions were added in HEK293 cells with or without the addition of 1μM DEX or DMSO/EtOH for 45 min. Western blot analysis then followed for the evaluation of AMPKα and phosphorylated AMPKα protein levels at Thr172. As shown in Figure 5, Mastiha fractions caused insignificant changes in AMPKα protein levels, while induction in phosphorylated AMPKα protein levels was observed. The medium-polar fraction caused approximately a 5-fold increase in the phosphorylated AMPKα level, while an approximately 1.5-fold increase was also observed by the polar fraction. Co-administration of the medium-polar with DEX suppressed the medium-polar-induced activation in AMPKα phosphorylation, possibly due to an antagonistic effect of DEX on medium-polar fraction activity. Interestingly, co-administration of DEX with the polar fraction had, in the end, an additive effect of enhancing the AMPKα phosphorylated level (Figure 5).

### 3.4. Chios Mastiha Fractions Suppressed the TNFα-Induced NF-κΒ Transcriptional Activation

**Figure 6:**
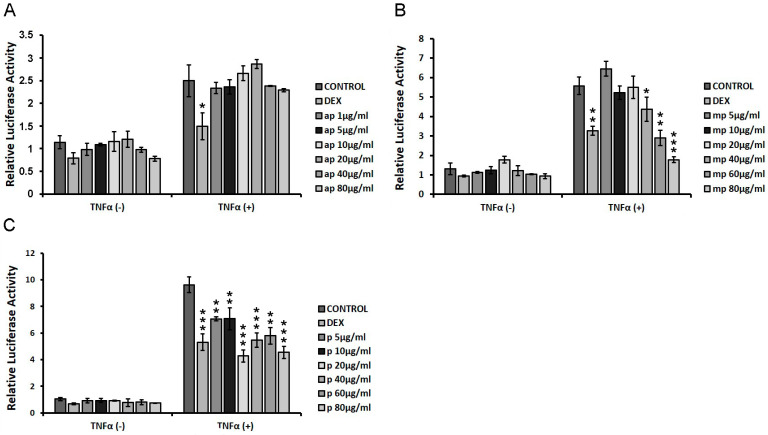
Chios Mastiha different polarity fractions suppressed the TNFα-induced NF-κΒ transcriptional activation. NF-κB-Luciferase reporter gene assay was applied in HEK293 cells treated with 1 μM DEX or 1–80 µg/mL of (**A**) apolar (ap), (**B**) medium-polar (mp), and (**C**) polar (p) Mastiha fractions and/or 20 ng/mL TNFα for 6 h. DMSO (1:1000) and EtOH (1:1000) were added in control condition. Relative luciferase activity results from normalization of luciferase activity to β-galactosidase activity. Relative luciferase activity of control cells was set as 1. Data are expressed as the mean ± SD, (n = 2–6), * *p* < 0.05; ** *p* < 0.01; *** *p* < 0.001.

HEK293 cells were transiently co-transfected with NF-κΒ-RE-luciferase and β-galactosidase constructs as described in the Materials and Methods section, and they were subsequently treated with increasing concentrations of apolar (ap), medium-polar (mp), and polar (p) fractions from Chios Mastiha (1 μg/mL–80 μg/mL), with or without the addition of 20 μg/mL TNFα for 6 h. Applying luciferase reporter gene assay, the effect of the fractions on NF-κΒ transcriptional activation was evaluated to assess the potential of Mastiha fractions regarding anti-inflammatory actions. As shown in Figure 6A, the apolar fraction showed no statistically significant reduction in NF-κΒ transcriptional activation, both in the absence and presence of TNFα, as compared with control DMSO/EtOH-treated cells. The medium-polar fraction suppressed the TNFα-induced NF-κΒ transcriptional activation by approximately 20–70% at concentrations of 40–80 μg/mL, being the most active one (Figure 6B), while the polar fraction caused a statistically significant reduction by approximately 25–50% at concentrations of 5–80 μg/mL (Figure 6C).

**Figure 7:**
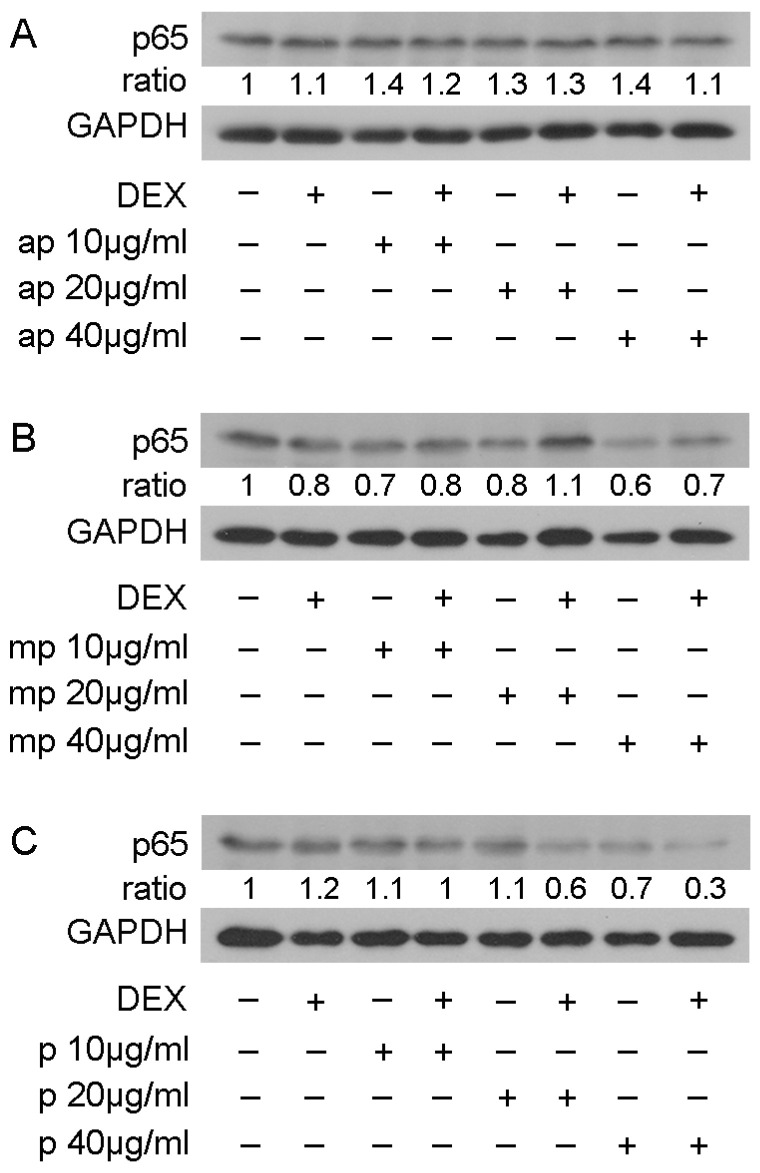
Regulation of p65 protein levels by Chios Mastiha different polarity fractions. Representative images from Western blot analysis of p65 in HEK293 cell extracts treated with 10, 20, and 40 μg/mL of apolar (**A**), medium-polar (**B**), and polar (**C**) fractions for 48 h (**B**). Ratios result from normalization of p65 bands intensity to the respective GAPDH ones. Relative protein levels in control cells were set as 1.

To examine whether the reduction in NF-κΒ transcriptional activation was linked with the reduction in its protein levels, Western blot analysis of the p65 subunit of NF-κΒ was applied inHEK293 cell extracts treated with 10, 20, and 40 μg/mL of Mastiha fractions, with or without 10 nM DEX or DMSO/EtOH for 24 h (Figure S9) and 48 h (Figure 7). Interestingly, the 24 h treatment of HEK293 with the Chios Mastiha fractions did not remarkably alter the p65 protein levels (Figure S9), whereas upon 48 h of treatment, the apolar fraction caused a 30–40% increase in the p65 protein levels at concentrations from 10 to 40 μg/mL (Figure 7A). A total of 40 μg/mL of medium-polar and polar resin fractions, meanwhile, caused a decrease up to 40% and 30%, respectively (Figure 7B,C).

### 3.5. Chios Mastiha Fractions Effect on Apoptotic Pathways

**Figure 8:**
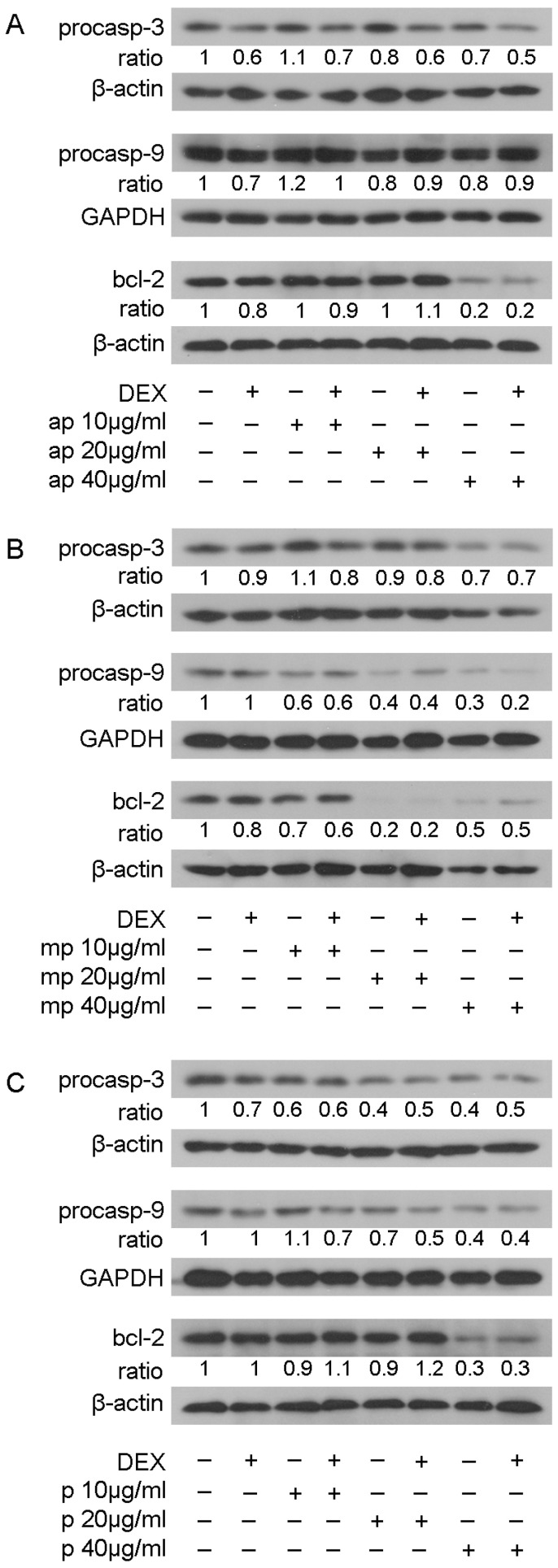
Regulation of procaspase-3, procaspase-9, and bcl-2 protein levels by Chios Mastiha different polarity fractions. Representative images of Western blot analysis of procaspase-3, procaspase-9, bcl-2, GAPDH, and β-actin in HEK293 cell extracts, precultured in hormone-free medium, and subsequently subjected to treatment with 10, 20, and 40 μg/mL of apolar (**A**), medium-polar (**B**), and polar (**C**) fractions for 48 h. Ratios result from normalization of the apoptosis-related protein bands intensity to the respective bands intensity of β-actin or GAPDH. Relative protein levels in control cells were set as 1.

The effect of resin fractions from *Pistacia lentiscus* L. *var. chia* on apoptotic mechanisms was examined by applying Western blot analysis of apoptotic and anti-apoptotic molecules, such as procaspase-3, procaspase-9, and bcl-2 in HEK293 cell extracts treated with 10, 20, and 40 μg/mL of Mastiha fractions with or without 10 nM DEX or DMSO/EtOH for 24 h (Figure S10) and 48 h (Figure 8). No remarkable changes were caused by Mastiha fractions in procaspase-3 protein levels upon 24 h treatment (Figure S10). However, 48 h treatment (Figure 8) with apolar, medium-polar, and polar resin fractions caused a dose-dependent reduction by up to 30%, 30%, and 60%, respectively, in procaspase-3 protein levels. Moreover, a dose-dependent reduction in procaspase-9 protein levels by 40% to 70% and by 30% to 60% was observed upon treatment with the medium-polar and the polar fractions (Figure 8B,C), respectively. Treatment with the apolar fraction caused a reduction in procaspase-9 protein levels up to 20% at a concentration of 40 μg/mL (Figure 8A). Consistently, the medium-polar fraction caused a remarkable reduction in bcl-2 protein levels by approximately 80%, even at a concentration of 20 μg/mL (Figure 8B). The apolar and polar fractions also reduced bcl-2 protein levels by approximately 80% and 70%, respectively, at a concentration of 40 μg/mL (Figure 8A,C).

## 4. Discussion

Chios Mastiha is widely used in traditional cooking as a food additive and flavoring, in beverages, and in many other applications such as perfumery, dentistry, and cosmetics [1,2]. More interestingly, the biological actions of Chios Mastiha have driven the attention of many research groups in recent years. A wide variety of anti-microbial [3,32,33,34], anti-oxidant [4,35,36], anti-inflammatory [9,37,38,39], anti-hyperlipidemic [40,41,42,43], anti-hyperglycemic [41,44], and anti-cancer [45,46,47] actions have emerged through in vitro and in vivo experiments, and through clinical trials, too. These biological actions are proposed to be attributed to Chios Mastiha bioactive substances, such as triterpenes [3,6,7,8,48], β-myrcene [1], phenolics [9], arabino-galactan proteins, neutral sugars, and uronic acids [49].

Triterpenes exhibit structural similarities with GCs [16]. Dexamethasone and other synthetic GCs have been widely used as strong anti-inflammatory and immunosuppressive drugs to cure various inflammatory bowel diseases and autoimmune diseases as well as blood malignancies due to their cell type specific apoptotic activities. Anti-inflammatory actions are exerted via GCs binding to the glucocorticoid receptor, the activation of its nuclear translocation, and, mainly, via activation of the transrepressional activities of the receptor on NF-κB activation [50]. Synthetic glucocorticoids also induce GR transcriptional activation, which is involved, among others things, in catabolism activation and glucose synthesis. Thus, the high dose and long-term administration of triterpenes for therapeutic purposes can lead to severe side effects, such as hyperglycemia, diabetes development, glaucoma, muscle atrophy, osteoporosis, and hypertension [51]. To minimize the adverse side effects of GCs, researchers have focused on the modulation of glucocorticoids molecules, or investigated selective glucocorticoid receptor agonists (SEGRAs) and the development of delivering systems to enhance the anti-inflammatory actions of GC-like molecules with reduced side effects [52]. In this context, triterpenes of plant origin have been revealed as promising lead molecules for the development of glucocorticoid receptor activators [16,17,18,19,20,53].

In this study, different polarity resin fractions from *Pistacia lentiscus* L. *var. chia* (Chios Mastiha) were investigated regarding their chemical composition, anti-proliferative, anti-inflammatory, apoptotic actions, and interference with GR signaling. Studies in the past have identified keto-oleanolic aldehyde, oleanolic aldehyde, lupeol, 24Z-masticadienonic acid methyl ester, and 24Z-isomasticadienonic acid methyl ester and phenolic compounds as components of Mastiha tree resin [3,7,9,48,54]. In this study, in an effort to characterize the active compounds of Mastiha, subfractionation of acetic Mastiha extract was performed to obtain three different polarity fractions––apolar, medium-polar, and polar. Phytochemical analysis revealed that the apolar and especially the medium-polar Mastiha fraction were rich in triterpenes, such as keto-oleanolic aldehyde, oleanolic aldehyde, lupeol, 24Z-masticadienonic acid methyl ester, and 24Z-isomasticadienonic acid methyl ester. The sticky polymer 1,4-poly-β-myrcene of Mastiha [3,7,48,54] was also identified by ^1^H NMR analysis to be present in the apolar Mastiha fraction, as expected. A subfraction of phenolic compounds was also achieved, since ^1^H NMR analysis revealed plenty of phenolic compounds and a lack of triterpenes in the polar Mastiha fraction.

The characterization of the biological activities of the isolated fractions was also assessed. Due to triterpenes enriching Chios Mastiha, we focused on the potential cross-talk of resin fractions with GR signaling. We showed for the first time that medium-polar and polar fractions from Chios Mastiha suppressed the DEX-induced GR transcriptional activation in HEK293 cells in a dose-depended manner (Figure 3C,D). This action could be attributed to the enrichment of the medium-polar and the polar fraction in steroid-like compounds. Steroid-like triterpenes were also found in the apolar fraction (Figure 1B,C). Nevertheless, the apolar fraction had minimal effect on GR transcriptional suppression. This effect may be attributed to the presence of the sticky polymer 1,4-poly-β-myrcene, which is proposed to hold together Mastiha’s triterpenes, reducing their bioavailability and minimizing their biological effects [1].

Reduction in GR transcriptional activation by the polar Mastiha fraction was accompanied by a decrease in GR protein levels (Figure 4C). Thus, we may hypothesize that a reduction in GR transcriptional activation may be due to the polar fraction-induced reduction in GR protein levels. This hypothesis is in accordance with our previous observations, revealing that different polarity leaves fractions from *Pistacia lentiscus* L. *var. chia* and can cause a dose-dependent suppression of the DEX-induced GR transcriptional activation and a reduction in GR protein levels in HEK293 cells, with the medium-polar and the polar fractions showing the highest activity. The latter activity was attributed to the leaves fractions inducing proteasomal activation [20]. Reduction in steroid receptors transcriptional activity by Chios Mastiha have also been observed in LNCaP and PC-3 human prostate cancer cells, where a reduction in androgen receptor (AR) transcriptional activation, inhibition of the AR binding to AR-response elements, and a reduction in AR protein levels in a dose-depended manner by Chios Mastiha was documented [55]. A reduction in GR protein levels was also observed by the apolar fraction, where triterpenes were also detected. Thus, our findings uncovered a possible common action of Chios Mastiha on the suppression of steroid receptor activity and the regulation of their protein levels, which could be attributed to steroid-like components, triterpenes, and phenolic compounds.

In accordance with the reduction in GR transcriptional activation, Mastiha fractions affect PEPCK and PPARα protein levels, which constitute GR target genes [27,28]. Specifically, Mastiha fractions, especially the medium-polar and the apolar ones enriched in triterpenes, caused a reduction in PEPCK protein levels (Figure 4A,B). The reduction in PEPCK protein levels by the medium-polar fraction is in accordance with its suppressive effect on the regulation of GR transcriptional activation. This action could take place in parallel to the proposed masticadienonic acid and isomasticadienonic acid interference with glucocorticoid receptor signaling via inhibition of 11β-hydroxysteroid dehydrogenase 1, an enzyme which converts inactive cortisone to active cortisol, leading to reduced cortisol levels and a subsequent hypoglycemic effect [56]. At the same time, the reduction in PEPCK protein levels by the apolar fraction is in accordance with the apolar fraction-induced reduction in GR protein levels. PEPCK is a key regulatory enzyme in glucose metabolism, as it catalyzes the first step in gluconeogenesis. Overexpression of PEPCK gene or overactivation of the PEPCK enzyme can result in hyperglycemia and the development of diabetes [57]. Moreover, previous studies have also shown that healthy volunteers and diabetic mice receiving Chios Mastiha on a daily basis had lower blood glucose levels and reduced insulin resistance [41,44,58]. Our findings confirm the observations above regarding the anti-hyperglycemic and anti-diabetic actions of Chios Mastiha––contributing to the characterization of the biochemical mechanism that this action takes place, and uncovering novel mechanisms of interference of Mastiha with the glucocorticoid receptor signaling via triterpenoids-induced regulation of GR transcriptional activation, regulation of GR protein levels, and/or GCs synthesis.

PPARα is also a GR target and plays an important role as a regulator of lipid metabolism [59]. PPARα induces gene expression of the proteins and the enzymes regulating mitochondrial fatty acids importation and β-oxidation, leading to ATP production. However, the administration of prolonged PPARα agonists in mice has caused an induction of many genes involved in the lipid biosynthetic pathway [60]. Moreover, overexpression of the PPARα gene in the hearts of diabetic mice has been related to both cardiac dysfunction and diabetic cardiomyopathy (DCM) development [59]. Previous studies suggested the anti-hyperlipidemic actions of Chios Mastiha: healthy volunteers receiving Chios Mastiha had lower total cholesterol, LDL, triglycerides, apolipoprotein A-1, apolipoprotein B, and lipoprotein A levels [42,44,58]. In this study, we showed the reduction in PPARα protein levels by Mastiha fractions. The medium-polar fraction did not cause a significant reduction in GR protein levels and also did not show a reduction in PPARα protein levels (Figure 4B), whereas the apolar and the polar fractions that induced a reduction in GR protein levels also induced a reduction in PPARα (Figure 4A,C), indicating that the reduction in the PPARα protein level by Mastiha fractions may be exerted, at least in part, through the regulation of GR protein levels. Overall, the reduced PEPCK and PPARα protein levels by Mastiha fractions come in accordance with the previously reported anti-hyperglycemic, anti-hyperlipidemic, and cardioprotective actions of Chios Mastiha. Our results indicate that anti-hyperglycemic action, via reduction in PEPCK protein levels, and GR transcriptional regulation may be exerted via triterpenoids present in the medium-polar and/or the apolar fractions, whereas antilipidemic action is possibly exerted via triterpenoids and phenolic compounds, too.

Moreover, Chios Mastiha medium-polar and polar fractions induced an increase in AMPKα phosphorylation at Thr172, while no notable changes in AMPK protein levels were induced (Figure 5). AMPK constitutes a major cellular energy sensor, regulating ATP/AMP levels. When ATP levels are decreased and AMP levels are increased, AMPK is phosphorylated at Thr172 and is activated by upstream kinases. Activated AMPK, then, promotes catabolic pathways and inhibits the anabolic ones to produce ATP and restore cellular energy levels [29,61]. Previous studies proved that dexamethasone and corticosterone treatment increase AMPK activity in HepG2 cells and rat liver, respectively [62]. In this study, we verified the dexamethasone-induced AMPKα phosphorylation at Thr172 in kidney HEK293 cells, and we uncovered similar actions by the medium-polar and the polar Mastiha fractions (Figure 5). Considering that both the liver and the kidney are gluconeogenic tissues, a regulatory mechanism of action of Chios Mastiha in glucose metabolism is further substantiated. Phosphorylated and activated AMPK could also regulate GR transcriptional activation through indirect GR phosphorylation [31]. Thus, induction in AMPK Thr172 phosphorylation may be linked, at least in part, to the suppression of GR transcriptional activation and, subsequently, to reduced PEPCK protein levels. Thus, the reduced PEPCK protein levels and the activation of AMPK, actions that are activated mainly by the medium-polar fraction, indicate that Chios Mastiha via triterpenoids components inhibits anabolic pathways and induces the catabolic ones to produce ATP.

The medium-polar fraction exhibited the highest anti-inflammatory actions among the three different polarity Mastiha fractions, too, as indicated by the suppression of the TNFα-induced NF-κΒ transcriptional activation in a dose-dependent manner and the reduction in the p65 NF-κΒ subunit. These effects may be attributed to the medium-polar enrichment in triterpenes, such as lupeol, 24Z-masticadienonic acid methyl ester, and 24Z-isomasticadienonic acid methyl ester. Lupeol is known to exhibit a wide range of anti-inflammatory activities, as indicated by both in vitro and in vivo studies. Lupeol reduces inflammation in arthritic and asthmatic mouse models and decreases the levels of proinflammatory cytokines, such as TNFα, IL-4, IL-5, and IL-13. Interestingly, lupeol has been found to exhibit equal anti-inflammatory activity to dexamethasone [63]. An additional proposed mechanism of the anti-inflammatory activity of lupeol in human intestinal epithelial cells also involves lupeol-induced suppression of the NF-κΒ inhibitor IκΒα phosphorylation and degradation [64]. The high anti-inflammatory activity of the medium-polar fraction indicates possible anti-inflammatory activity of the 24Z-masticadienonic acid methyl ester and the 24Z-isomasticadienonic acid methyl ester, too, through the regulation of NF-κΒ activity.

The apolar fraction showed anti-inflammatory activity, as well, albeit less than the medium-polar one, possibly due to the presence of the sticky polymer 1,4-poly-β-myrcene and/or to a different composition of triterpenoids. Anti-inflammatory activity of the whole extract from Chios Mastiha powder has been previously reported. Thus, it has been observed that Chios Mastiha powder reduced the serum TNFα levels in male rats with gastric mucosal inflammation [38]. Moreover, it has been shown that Chios Mastiha neutral extract suppressed the phosphorylation of p65 subunit of NF-κΒ in TNFα-stimulated human aortic endothelial cells (HAEC) [39]. AMPK is also proposed to suppress inflammation via inhibiting the signal transducer, the activator of Transcription-1, and NF-κΒ signaling [65,66]. Thus, data from our study is in accordance with previous findings positing the anti-inflammatory actions of Chios Mastiha, but we, for the first time, provide evidence that the most active compounds for these actions are the triterpenoids present in the medium-polar fraction, and that these actions may be exerted via interference of terpenoids with GR and AMPK signaling.

Apoptotic and anti-proliferative activities of Chios Mastiha have been previously demonstrated. Chios Mastiha inhibited the proliferation of human colon cancer HCT116 cells in a dose-dependent manner [67]. In the above cells, Chios Mastiha increased the activity of caspase-3, -8, and -9, inducing cell apoptosis [68]. Chios Mastiha also caused a reduction in the anti-apoptotic bcl-2 protein levels and increased the pro-apoptotic bax protein levels in human pancreatic carcinoma cell lines BxPC-3 and COLO357 [69]. In this study, we showed that Chios Mastiha fractions inhibited the cell viability of HEK293 in a dose-dependent and time-dependent manner, with the medium-polar fraction being the most cytotoxic one while the apolar fraction caused less cytotoxicity (Figure 2). The induction of apoptosis by the Mastiha fractions, as indicated by the reduction in the procaspase-3, procaspase-9, and bcl-2 protein levels in a dose-dependent manner, was also observed. The reduction in procaspase-9 indicates mitochondrial-dependent induction of apoptosis. The medium-polar fraction was the most active one. The presence of lupeol in the medium-polar fraction may be responsible for these actions. It has previously been shown that lupeol inhibits cell proliferation by inducing cell cycle arrest via decreasing the expression of cyclins and cyclin-dependent kinases (CDKs) [63,64]. Moreover, lupeol has been widely studied for its anticancer and apoptotic activities. It has been found that lupeol induces cell apoptosis through the activation of the mitochondrial pathway. Lupeol, in particularly, increases the levels of proapoptotic bax protein, decreases the levels of anti-apoptotic bcl-2 protein, and, therefore, activates caspases cascades [64]. Masticadienonic acid also present in the medium-polar fraction has previously been shown to induce apoptosis and inhibit cell growth in prostate cancer cells [70]. Our previous studies revealed that different polarity leaves fractions from *Pistacia lentiscus* L. *var. chia* caused a reduction in procaspase-3, procaspase-9, and bcl-2 protein levels in HEK293 cells [20]. Thus, our results for the first time have revealed that different polarity Chios Mastiha fractions have a differential effect on the induction of apoptosis, which is exerted by the mitochondrial intrinsic pathway.

## 5. Conclusions

To sum up, Chios Mastiha different polarity fractions inhibited the cell viability of HEK293 in a dose and time-dependent manner, possibly by activating the mitochondrial dependent apoptotic pathway. Moreover, Chios Mastiha medium-polar and polar fractions suppressed the GR and NF-κΒ transcriptional activation, and caused a reduction in the PEPCK and p65 protein levels, substantiating their potential anti-hyperglycemic and anti-inflammatory activities. These actions may be associated with the effect of the medium-polar and the polar fractions on AMPKα phosphorylation and activation, which subsequently could directly or indirectly interfere with GR signaling. Medium-polar enrichment in triterpenes and especially in lupeol, 24Z-masticadienonic acid methyl ester, and 24Z-isomasticadienonic acid methyl ester that show structural similarities with GCs indicates that these three compounds are possibly the bioactive compounds responsible for the interference of Mastiha fractions with the GR signaling, leading to Mastiha’s anti-glycemic and anti-inflammatory actions. This hypothesis is interesting and needs further investigation. Moreover, the reduction in GR transcriptional activation by the polar fraction may be attributed to the polar fraction’s suppressive effect on GR protein levels, which lead to the reduction in PEPCK and PPARα protein levels, and which may be associated with the anti-hyperglycemic and anti-hyperlipidemic activities of Mastiha. The apolar Mastiha fraction had less effect on GR and NF-κΒ transcriptional suppression, possibly due to the presence of the polymer 1,4-poly-β-myrcene, which hinders the maximum biological effect of the Mastiha’s bioactive triterpenes and/or to the differential triterpene composition compared to the medium-polar fraction. Thus, the interference of Mastiha triterpenes with glucocorticoid, NF-κB, and AMPK signaling is proposed to be responsible for its anti-hyperglycemic, ani-hyperlipidemic, and anti-inflammatory activities highlighting its potential applications in many fields of pharmaceutical, cosmetic, and nutraceutical interest.

## Data Availability

All data, tables, and figures are original. Details on data analysis are available from the corresponding author upon reasonable request.

## Supplementary Materials

The following supporting information can be downloaded at: https://www.mdpi.com/article/10.3390/foods12071390/s1, Supplementary Data: Figure S1: ^1^H NMR of 1,4-poly-β-myrcene in CDCl_3_ 400 MHz; Figure S2: ^1^H NMR of keto-oleanolic aldehyde in CDCl_3_ 400 MHz; Figure S3: ^1^H NMR of oleanolic aldehyde in CDCl_3_ 400 MHz; Figure S4: ^1^H NMR of lupeol in CDCl_3_ 400 MHz; Figure S5: ^1^H NMR of masticadienonic acid methyl ester in CDCl_3_ 400 MHz; Figure S6: ^1^H NMR of isomasticadienonic acid methyl ester in CDCl_3_ 400 MHz; Figure S7: ^1^H NMR of polar fraction in CDCl_3_ 400 MHz; Figure S8: Effect of Chios Mastiha fractions on PEPCK and PPARα protein levels upon 24 h treatment; Figure S9: Effect of Chios Mastiha fractions on p65 protein levels upon 24 h treatment; Figure S10: Effect of Chios Mastiha fractions on procaspase-3 protein levels upon 24 h treatment.
