# Supplementary material for: Regulation of Energy Metabolism and Anti-Inflammatory Activities of Mastiha Fractions from Pistacia lentiscus L. var. chia
Source: Foods. 2023 Mar 24;12(7):1390. doi: 10.3390/foods12071390 (PMC10093696; doi:10.3390/foods12071390)
Supplement: Supplementary file 1 [file foods-12-01390-s001.zip › foods-2206641-supplementary.pdf]

Supplementary Data

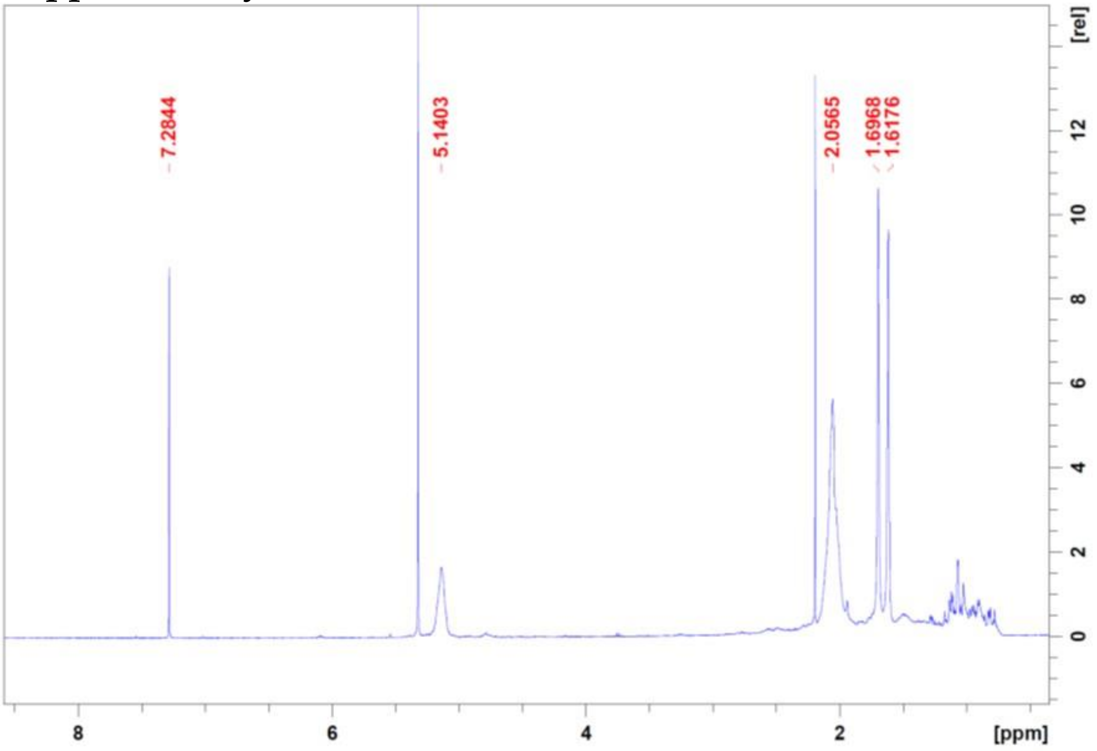

Figure S1: ^{1}H NMR of 1,4-poly-β-myrcene in $CDCl_3$ 400 MHz.

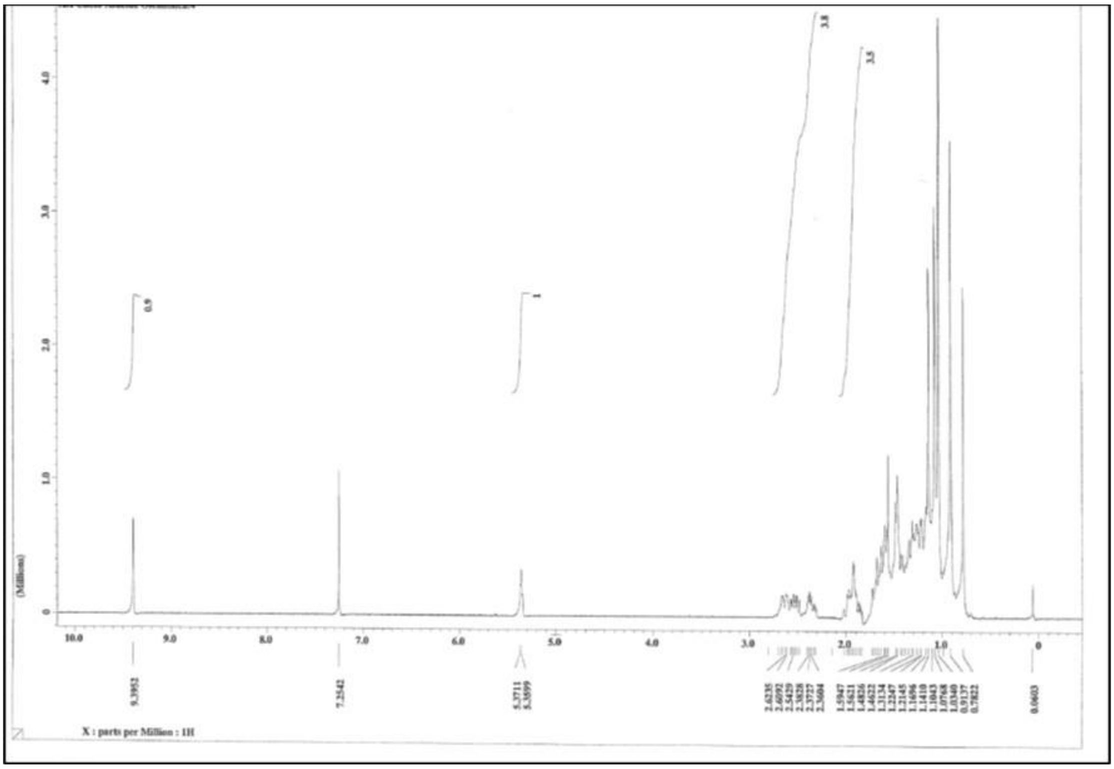

Figure S2: ^{1}H NMR of keto-oleanolic aldehyde in $CDCl_3$ 400 MHz.

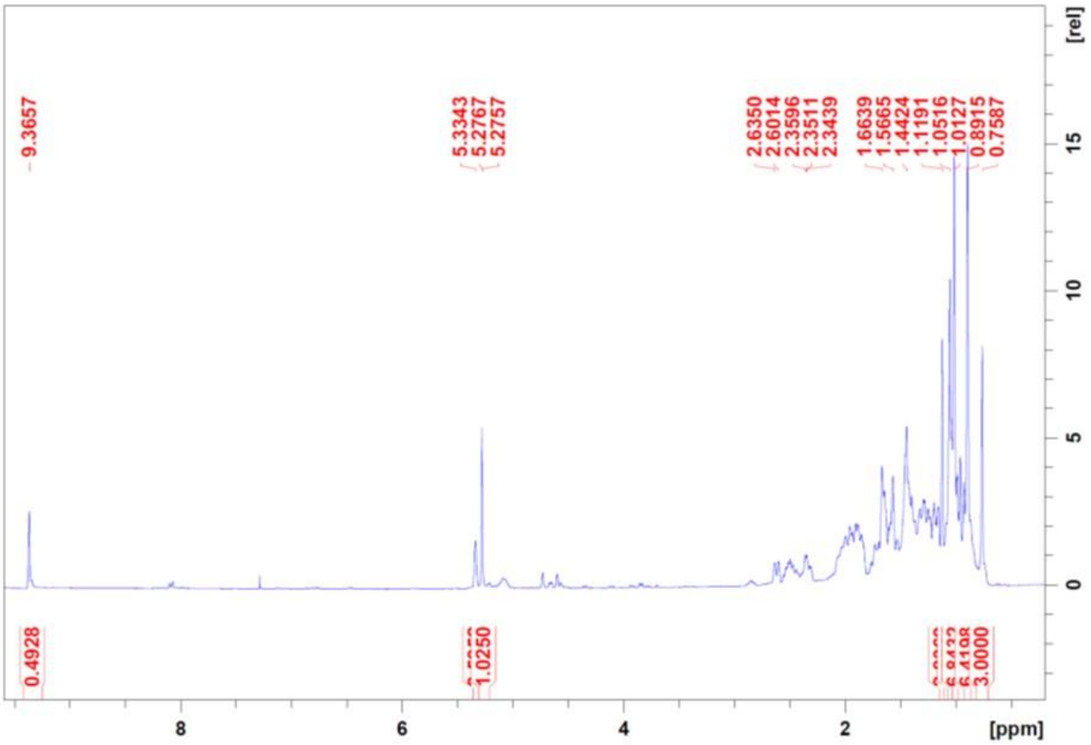

Figure S3: ^{1}H NMR of oleanolic aldehyde in $CDCl_3$ 400 MHz.

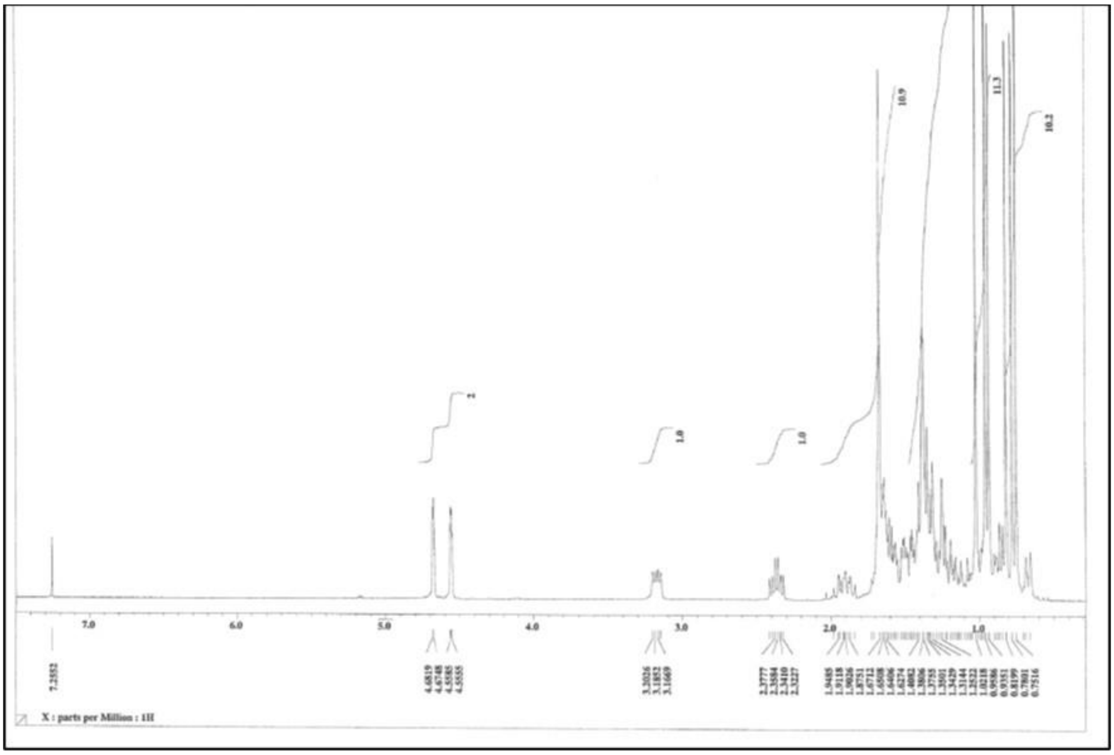

Figure S4: ^{1}H NMR of lupeol in $CDCl_3$ 400 MHz.

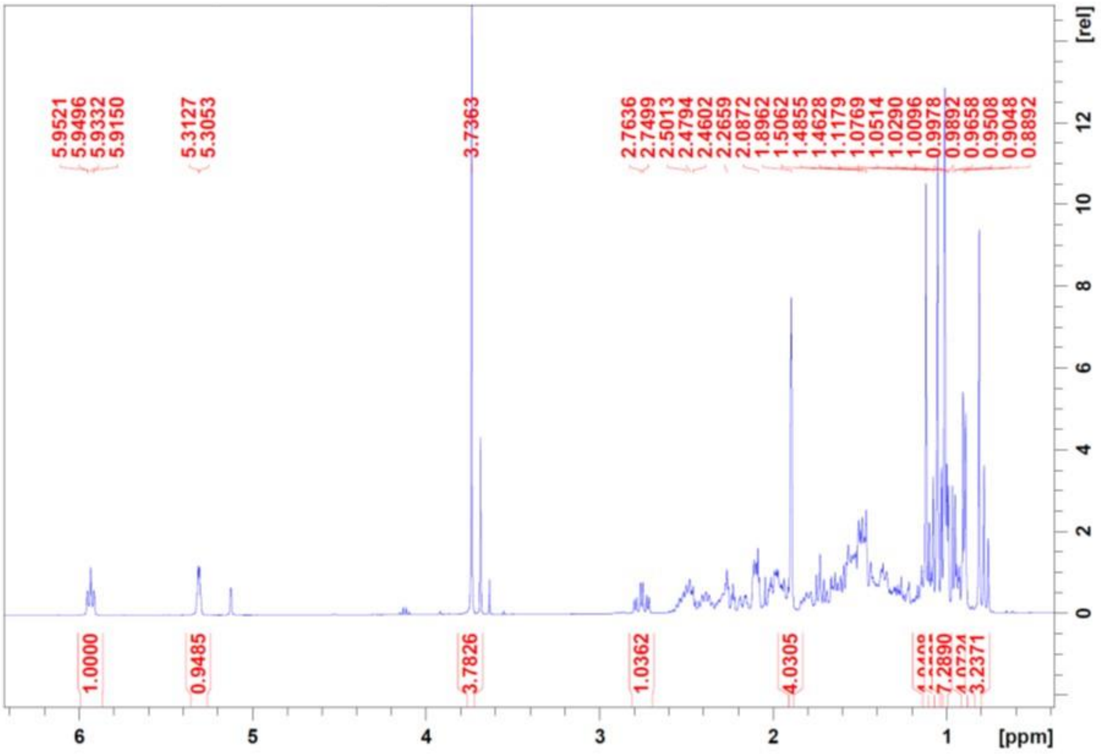

Figure S5: ^{1}H NMR of masticadienonic acid methyl ester in $CDCl_3$ 400 MHz.

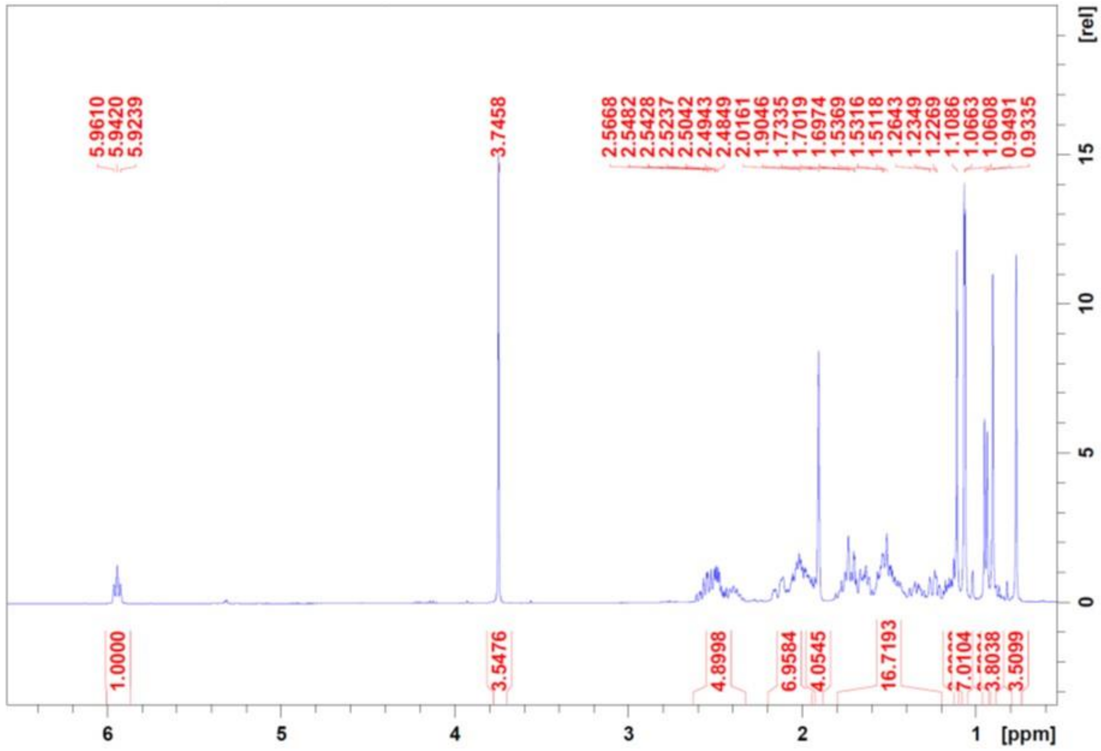

Figure S6: ^{1}H NMR of isomasticadienonic acid methyl ester in $CDCl_3$ 400 MHz.

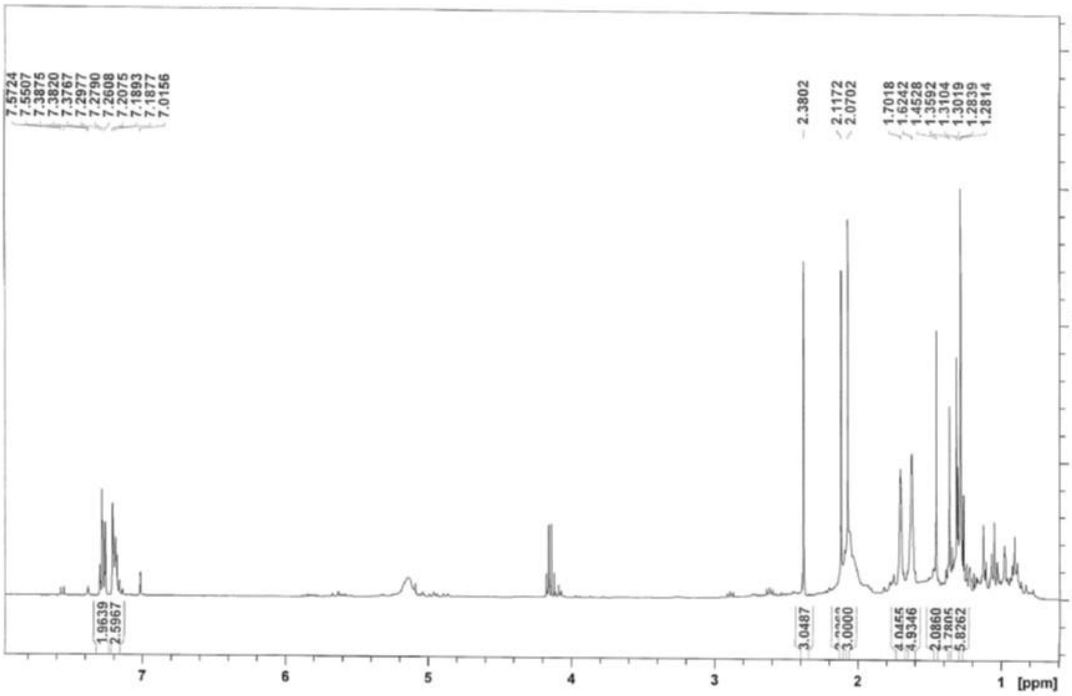

Figure S7: ^{1}H NMR of polar fraction in $CDCl_3$ 400 MHz.

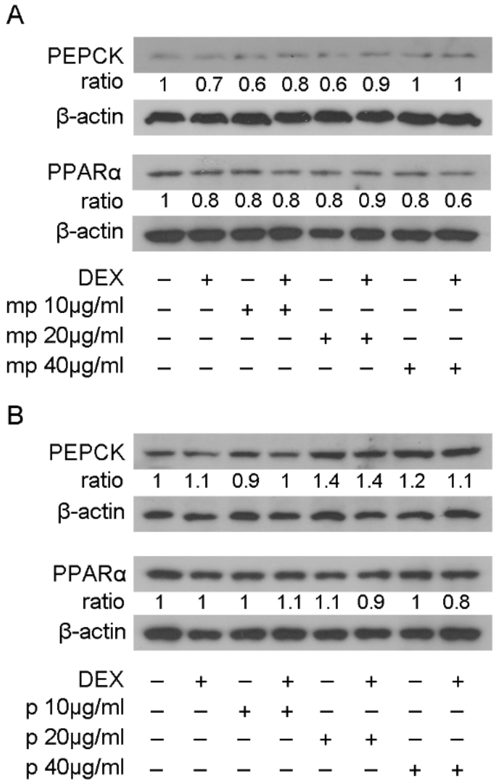

Figure S8: Effect of Chios Mastiha fractions on PEPCK and PPARα protein levels, upon 24h treatment. Western blot analysis of GR, PEPCK and PPARα protein levels was applying in HEK293 cell extracts treated with 10, 20 and 40μg/ml medium-polar (A) and polar (B) fractions for 24h. Ratios express normalization of bands intensity

compared with the respective β-actin one. Relative protein levels in control cells was set as 1.

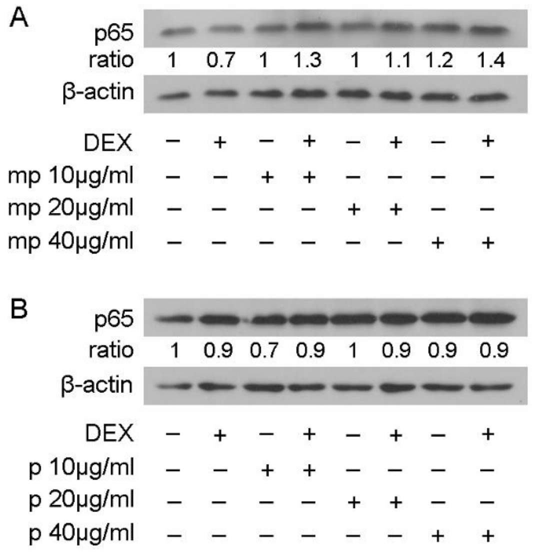

Figure S9: Effect Chios Mastiha fractions on p65 protein levels, upon 24h treatment. Western blot analysis of p65 protein levels was applying in HEK293 cell extracts treated with 10, 20 and 40μg/ml medium-polar (A) and polar (B) fraction for 24h. Ratios express normalization of bands intensity compared with the respective β-actin one. Relative protein levels in control cells were set as 1.

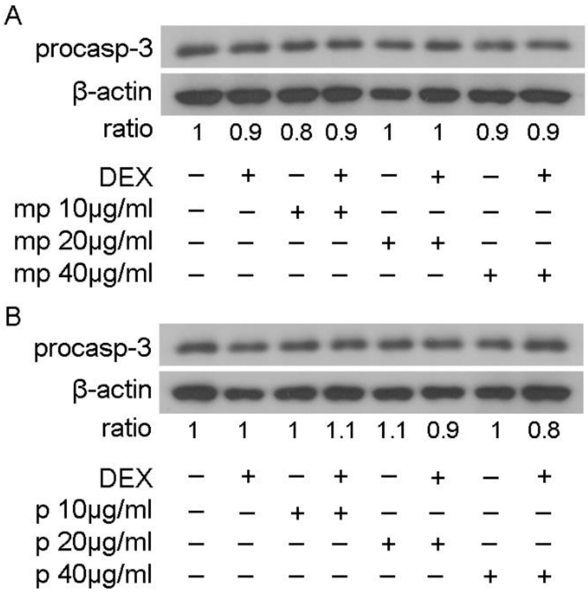

Figure S10: Effect of Chios Mastiha fractions on procaspase-3 protein levels, upon 24h treatment. Western blot analysis of procaspase-3 protein levels was applying in HEK293 cell extracts treated with 10, 20 and 40μg/ml medium-polar (A) and polar (B) fraction for 24h. Ratios express normalization of bands intensity compared with the respective β-actin one. Relative protein levels in control cells were set as 1.
